# Syntheses of Marine Natural Products via Matteson Homologations and Related Processes

**DOI:** 10.3390/md23010020

**Published:** 2025-01-02

**Authors:** Uli Kazmaier

**Affiliations:** 1 Organic Chemistry, Saarland University, Campus Building C4.2, D-66123 Saarbruecken, Germany; u.kazmaier@mx.uni-saarland.de; 2 Helmholtz Institute for Pharmaceutical Research Saarland (HIPS), Saarland University, Campus, C8.1, D-66123 Saarbruecken, Germany; 3 Pharma Science Hub, Saarland University, Campus, Geb. A2 3, D-66123 Saarbruecken, Germany

**Keywords:** boronic esters, borylation, lithiation, Matteson homologation, marine natural products

## Abstract

Matteson homologation, a successive extension of chiral boronic esters, is perfectly suited for the synthesis of complex molecular structures containing several stereogenic centers. The “classical version” allows the introduction of various functional groups in a 1,2-*anti*-configuration. The absolute configuration is determined by the choice of the chiral auxiliary, which can be used to introduce several stereogenic centers. In contrast, in Aggarwal’s lithiation-borylation strategy, new chiral auxiliary reagents must be used in each reaction step, which on the other hand allows the individual insertion of the desired stereogenic centers. Both methods have their individual advantages and disadvantages and are well suited for the synthesis of marine natural products.

## 1. Introduction

**Figure 1:**
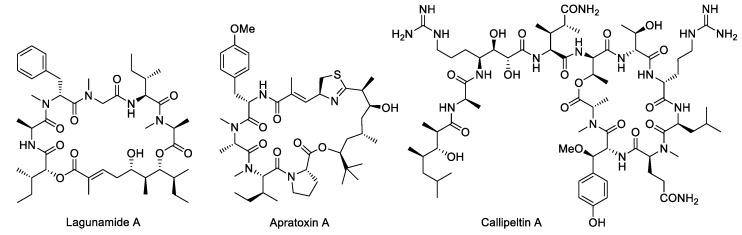
Marine cyclodepsipeptides.

Already in early human cultures, the biological activity of various natural products was recognized and used for different purposes [1]. The first records of the medicinal use of plants in China, India and the Middle East date back 5000 years [2]. Due to great advances in analytics, but at the same time also in organic synthesis, numerous active natural products have been isolated, identified and synthesized during the last century. In the years 1981–2006, for example, about 50% of all newly discovered active substances in the field of small molecules were substances derived from natural products [3]. Most of them were isolated from a wide range of plants, fungi and microorganisms [4], in which marine natural products represent a particularly important class of therapeutically interesting active ingredients [5,6,7]. The structures of the isolated compounds are as diverse as their producers. In addition to (cyclic) peptides and polyketides, also cyclodepsipeptides, which contain both structural elements, are well represented. Typical examples of these are lagunamide A [8], apratoxin A [9] or callipeltin A [10] (Figure 1).

However, isolation of bioactive substances often provides only insufficient quantities for biological studies or even complete structure elucidation, and thus the total synthesis of these compounds has been established as a valuable tool, not only to provide enough material for biological studies but often also for structure elucidation or verification [11]. There are a variety of methods for the synthesis of the often rather unusual amino acids [12,13,14], but asymmetric aldol reactions and related reactions are mainly used to build polyketide structures [15,16]. These methods are excellently suited for the formation of 3,5,7,… polyhydroxylated carboxylic acids, such as in lagunamide, but can only be used to a limited extent in the synthesis of polyketides with other, unusual substitution patterns, such as in apratoxin. In 1980 Matteson et al. reported an asymmetric homologation of boronic esters [17] which is also an efficient tool for total synthesis but is also much more flexible than aldol reactions with regard to the substitution pattern. In addition, not only suitable for the generation of polyketides but also for peptides and other substance classes. This review summarizes the applications of Matteson homologations and related reaction sequences [18] in the synthesis of marine natural products.

## 2. Homologations of Boronic Esters

### 2.1. Matteson Homologations

**Scheme 1:**
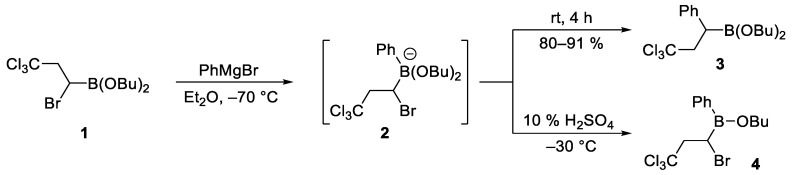
Nucleophilic substitution of α-bromoboronic esters according to Matteson et al. [19].

In 1963, Matteson et al. reported that the nucleophilic substitution of α-haloboronic esters is considerably facilitated by the neighboring group effect of the boron atom [19]. For example, when the α-bromoboronic acid ester **1** was reacted with phenylmagnesium bromide, the substitution product **3** was obtained in very good yield after warming the reaction mixture up to room temperature (Scheme 1). Aqueous acidic workup at low temperature, on the other hand, yielded the boric acid ester **4**, indicating that the reaction proceeded from **1** to **3** via the boronate complex **2**. From **2**, the substitution product **3** was formed via 1,2 migration of the phenyl group.

**Scheme 2:**
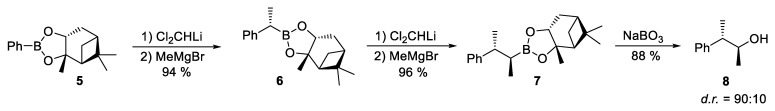
Matteson homologations using pinanediol as chiral auxiliary.

Initially, no suitable, generally applicable routes for the synthesis of α-haloboronic acid esters were available, and therefore, the method was seldom used until Matteson et al. in 1980 reported the homologation of boronic esters to α-chloroboronic esters by using deprotonated dichloromethane [20,21]. The α-chloroboronic esters could then be reacted with a variety of nucleophiles such as alcoholates [22], enolates [23,24], organolithium compounds or Grignard reagents [18,25]. If boronic esters of chiral diols were used, highly diastereoselective reactions to chiral α-chloroboronic esters became available. In a first report in 1980 Matteson et al. demonstrated that the pinanediol ester of phenylboronic acid **5** on reaction with (dichloromethyl)lithium provides the corresponding α-chloroboronic ester which could be reacted with MeMgBr, whereby the boronic ester **6** was obtained (Scheme 2) [17].

Repeated homologation and reaction with the same Grignard reagent yielded the boronic ester **7**, which was oxidized to alcohol **8** with a good diastereomeric ratio (*d.r.*). The pinanediol used as a chiral auxiliary can easily be obtained in both enantiomeric forms by *syn*-dihydroxylation of either (+) or (−)-α-pinene [26].

As has been shown, the addition of zinc chloride can significantly increase both the yields and the *d.r.* values of the Matteson homologation. With pinanediol as a chiral auxiliary, diastereoselectivities of up to 98.5–99.5% can be achieved under these conditions. By using chiral, C_2_-symmetric diols as auxiliaries, even better diastereoselectivities can be obtained. Excellent results can be achieved, for example, with diisopropylethandiol (DIPED) [27], which can be obtained from tartaric acid esters [28]. Dicyclohexylethandiol (DICHED) is so far the most used symmetric chiral diol, as it is relatively easy to synthesize from *trans*-stilbene [29,30].

As already illustrated with the example in Scheme 2, the Matteson homologation is perfectly suited for an iterative setup of adjacent stereocenters. The stereochemical outcome of the reaction is almost exclusively controlled by the chiral diol in a substrate-controlled manner, giving access to the 1,2-*anti*-configured product [18,25].

**Scheme 3:**
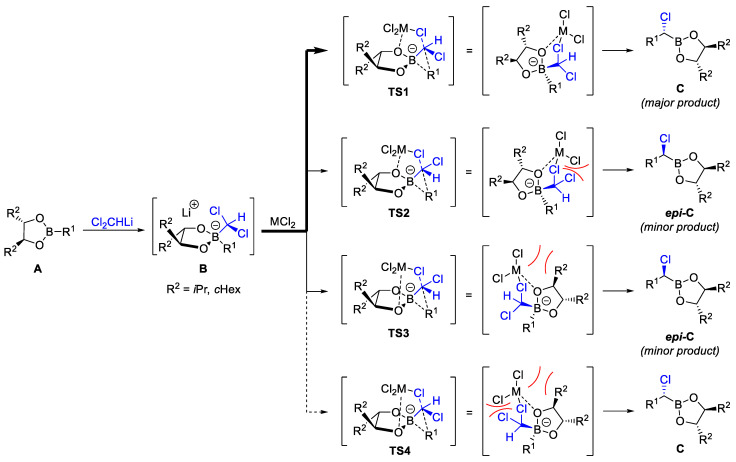
Mechanism of Matteson homologation.

The generally accepted mechanism of the Matteson homologation is shown in Scheme 3 [31]. In the first step, the carbenoid (dichloromethyl)lithium is added to the chiral boronic ester **A**, forming a tetrahedral boronate complex **B**. In the presence of zinc chloride, a 1,2-migration of the substituent R’ to either **C** or ***epi*-C** takes place. This rearrangement in principle can proceed via four different transition states (TS). In all cases, an oxygen atom of the diol and a chlorine atom of the former carbenoid coordinate with the Lewis acid. In the most favorable transition state **TS1**, the coordination of zinc takes place via the free electron pair on the oxygen, which is located *anti* to the residue R’. The chlorine atoms of the (dichloromethyl) residue arrange themselves in such a way that the uninvolved chlorine atom occupies the greatest possible distance from the zinc chloride, forming the main diastereomer **C**. The transition states **TS2** and **TS3**, which each lead to the minor diastereomer ***epi*-C**, are energetically much less favorable. In the case of **TS2**, the spatial proximity of the second Cl atom of the (dichloromethyl) residue to the zinc chloride and in the case of **TS3** the proximity of R to the Lewis acid leads to steric obstruction. Particularly unfavorable and therefore negligible is **TS4**, in which steric obstruction occurs at both positions. In 1998, Midland was able to confirm by ab initio calculations that **TS1** is the most favorable transitional state [32].

**Scheme 4:**
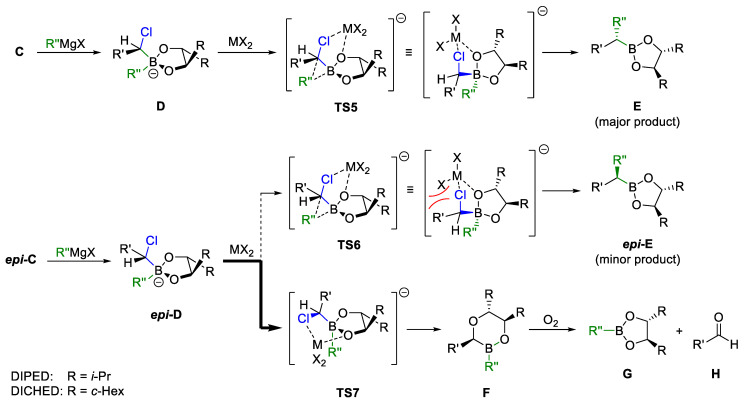
Sequential double diastereo-differentiation by using C_2_-symmetric diols as auxiliary according to Matteson et al. [33].

If **C** and ***epi*-C** are next reacted with a nucleophile, such as a Grignard reagent, the boronate complex **D** or ***epi-D*** is formed in analogy to the first step (Scheme 4). In the subsequent 1,2-rearrangement, which is catalyzed by zinc or magnesium salts (MX_2_), the chlorine atom and the newly introduced residue R″ arrange themselves antiperiplanar starting from **D** in the transition state **TS5** and the major diastereomer **E** is obtained. If C2-symmetric diols such as DICHED or DIPED are used as auxiliaries, this step further improves the diastereoselectivity, since an analogous 1,2-shift from ***epi*-D** to ***epi*-E** via the sterically hindered transition state **TS6** hardly takes place. In this case, the minor diastereomer ***epi*-D** preferentially reacts via 1,2-migration of an alkoxy residue of the chiral diol via **TS7** to dioxaborinan **F**, which is rapidly oxidized in air to the boronic ester **G** and the aldehyde **H**. Since **D** and ***epi*-D** react to different products, extremely high diastereoselectivities for the major product **E** can be achieved with such C_2_-symmetrical chiral diols [33].

The (dichloromethyl)lithium commonly used in Matteson homologations can be obtained by deprotonation of dichloromethane with *n*-butyllithium at −100 °C. The carbenoid solution has to be mixed with the boronic acid ester to be homologated [20,21]. An easier method to generate (dichloromethyl)lithium was developed by Brown et al. wherein the carbenoid is produced in situ in the presence of the boronic acid ester by deprotonation of dichloromethane with *sec*-butyllithium at −78 °C [34]. Alternatively, dichloromethane can also be deprotonated with LDA at −40 °C in situ, which is more suitable for reactions on a larger scale [35].

For some substitution reactions, α-chloroboronic esters are too unreactive. In these cases, the use of the appropriate α-bromoboronic esters, which can be obtained by homologation with (dibromomethyl)lithium, is recommended. This carbenoid can only be produced in situ by deprotonation with lithium diisopropylamide (LDA), as *n*-butyllithium preferentially undergoes a halogen-metal exchange to (bromomethyl)lithium [36]. α-Bromoboronic esters are more reactive than their chlorine analogs, but they are also more susceptible to epimerization, which should be taken into account in synthetic applications [37].

In addition to carbenoids of the X_2_CHLi type, (chloromethyl)lithium or (bromomethyl)lithium (XCH_2_Li) can also be used in Matteson homologations, whereby boronic esters can be extended by a CH_2_ group. The carbenoids can be generated in situ by halogen-metal exchange with *n*-butyllithium from chloroiodomethane [38], bromochloromethane [39] or dibromomethane [40]. Alternatively, hydrides can also be used as nucleophiles in Matteson homologations, allowing for a two-stage introduction of CH_2_ groups via reduction of the α-haloboronic esters. For this purpose, KBH(*i*Pr)_3_ [39], LiBHEt_3_ [41,42], NaH [43] or NaBH_4_ [44] can be used as hydride sources. The application of the corresponding deuterides enables the synthesis of asymmetrically deuterated compounds [45].

The Matteson reaction has the great advantage that with only one auxiliary any number of stereocenters can be introduced through successive homologation steps [46,47].

### 2.2. Reagent-Controlled Homologations

**Scheme 5:**
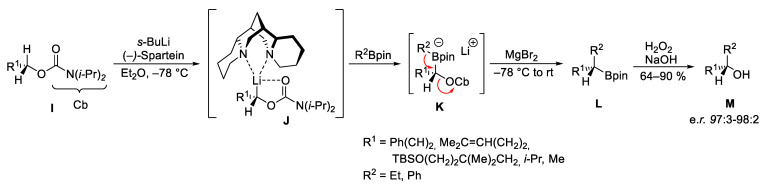
Lithiation-borylation of Hoppe carbamates according to Aggarwal et al. [50].

Although the Matteson homologation enables the highly diastereoselective construction of adjacent stereocenters in a 1,2-*anti*-arrangement, it is less suitable if the corresponding 1,2-*syn*-products are required. In these cases, either an exchange of the chiral diol [48] or, in the case of non-C_2_-symmetric diols as an auxiliary, an inversion of the α-stereocenter is necessary [49]. Alternatively, such structures can be obtained using a lithiation-borylation strategy according to Aggarwal et al. [50] In this reagent-controlled variant of the Matteson homologation, achiral pinacol boronic esters are reacted with Hoppe’s chiral lithiated carbamates (Cb) **I** [51,52]. The stereoselectivity in this case is determined by the configuration of the lithiated carbamate (**J**) (Scheme 5) [50], which is formed by deprotonation of a prochiral carbamate in the presence of a chiral auxiliary base, such as sparteine [53,54,55]. On reaction with a pinacol boronic ester, the borate complex **K** is formed, which delivers the elongated boronic ester **L** with good selectivity after MgBr_2_-catalyzed 1,2-shift and substitution of the carbamate residue. Boronic ester **L** can be used again as a substrate for further homologations or converted into the secondary alcohols **M** by oxidation. Alternatively, Beak’s 2,4,6-triisopropylbenzoyl ester (TIP) can be used as a directing group in such deprotonation reactions [56]. The TIB-esters are more electron-withdrawing and therefore the α-proton is slightly more acidic and easier to remove, which might be an advantage with critical substrates.

The Aggarwal homologation allows the generation of adjacent stereocenters in both *syn*- and *anti*-constellation. However, a major disadvantage of the reaction is that stoichiometric amounts of sparteine are required as a chiral auxiliary for each homologation step. (−)-Sparteine and (+)-sparteine occur naturally and surrogates [57] are also synthetically accessible, but the availability and thus the price has fluctuated greatly in the last two decades [58]. Nevertheless, this method was used in the synthesis of a number of natural products [59,60].

**Scheme 6:**
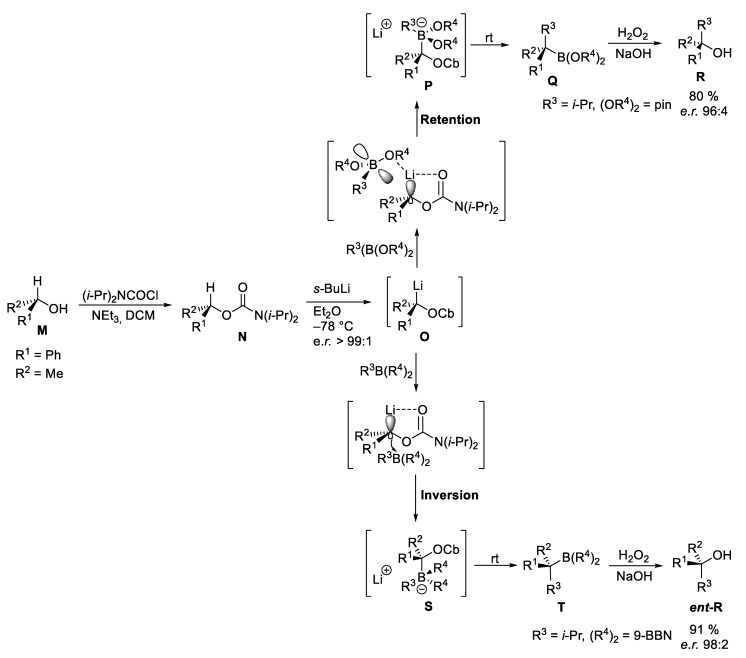
Lithiation-borylation of secondary Hoppe carbamates according to Aggarwal et al. [63].

In principle, the use of sparteine is not necessarily required if carbamates of secondary chiral alcohols **N** are used (Scheme 6). In 1990, Hoppe and coworkers showed that benzylic carbamates are particularly suitable for deprotonation, whereby the configuration at the chiral center is largely retained [61,62]. The lithiated species **O** can then be reacted with boronic esters with retention of the configuration to boronates **P**, which form the tertiary boronic esters **Q** with excellent chirality transfer via 1,2-shift of the residual R^3^. Subsequent oxidation leads finally to the chiral tertiary alcohols **R** [63].

Furthermore, the chiral carbenoids **O** were subjected to substitution with boranes, whereby the tertiary organoboron compounds **S** were formed. Interestingly, this substitution proceeded via boronate **T** under inversion of the original configuration. After oxidation of these, the corresponding enantiomeric tertiary alcohols *ent*-**R** were obtained with excellent e.r. [64,65].

The retention in the addition step to the borate complex **P** can be attributed to an additional complexation between the lithiated carbamate and one of the *O*-atoms of the boronic ester. The corresponding boranes do not have any complexing groups, so the attack takes place under inversion from the opposite face [63]. 

**Scheme 7:**
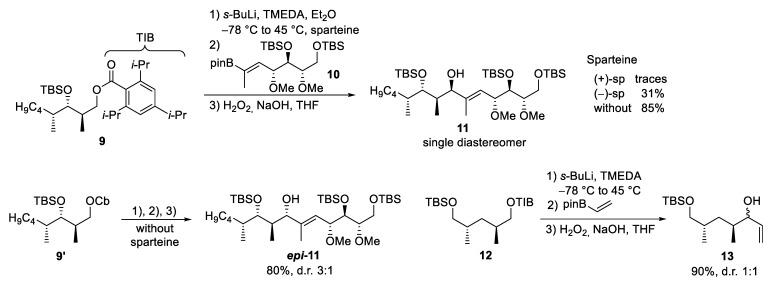
Sparteine-free homologation according to Kalesse et al. [66].

The fact that the use of sparteine is not necessarily helpful or necessary when using chiral primary esters or carbamates was shown by the work from the Kalesse group. During the synthesis of chondrochlorene, in which the chiral TIB-ester **9** had to be reacted with the vinylic boronic ester **10**, both (+)- and (−)-sparteine (sp) yielded the same product but in only very moderate yields, while without the addition of the ligand, an excellent yield of 85% and a single diastereomer could be obtained (Scheme 7) [66]. Interestingly, the Cb-carbamate **9′** provided the opposite diastereomer ***epi*-11**, albeit with poorer selectivity [67,68]. The stereogenic β-center seems to be crucial for the stereocontrolling effect of the substrate. If this substituent is missing as in **12**, the desired product **13** is formed in the sparteine-free version with an excellent yield but as a 1:1 diastereomer mixture. In contrast, in the presence of sparteine, the expected products are enantiomeric pure, but in this case, the stereochemical outcome of the reaction was solely controlled by the sparteine used.

**Scheme 8:**
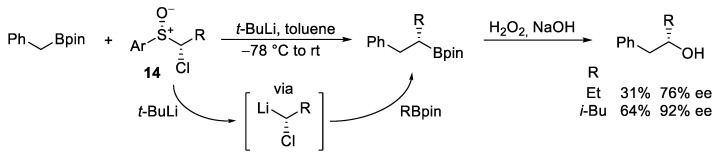
Sparteine-free homologation according to Blakemore et al. [69,70].

Blakemore et al. were the first to describe a sparteine-free “lithiation-borylation” reaction as early as 2007 (Scheme 8) [69,70]. However, their α-sulfinyl chlorides **14** require a two-step synthesis via enantioselective Jackson–Ellman–Bolm oxidation [71,72,73] and Yamakawa chlorination [74] and are less stable. Also, the yields and enantioselectivities are often only moderate [75,76,77,78,79].

### 2.3. Catalytic Homologations

**Scheme 9:**
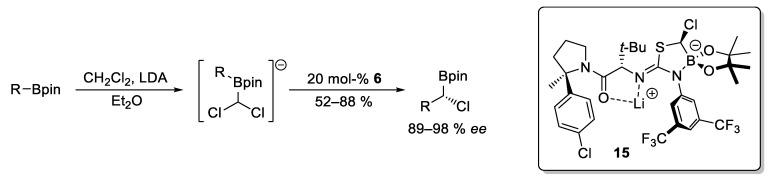
Catalytic, enantioselective homologation according to Jacobsen et al. [82,83,84].

A catalytic, enantioselective version of the Matteson homologation could circumvent the disadvantages of both methods. However, the first experiments on the homologation of pinacol boronic esters with chiral ytterbium catalysts were only successful in moderate enantioselectivities and required high catalyst loadings [80,81]. Jacobsen and coworkers achieved significantly better results with the lithium-isothiourea-borate complex **15** [82,83,84]. With this organocatalyst, various pinacol boronic esters could be homologated to α-chloroboronic esters via the corresponding boronate complex with good yield and enantioselectivity (Scheme 9). The α-chloroboronic esters could then be subjected to further homologations in the usual manner.

## 3. Syntheses of Marine Natural Products via Matteson Homologation

The homologation of boron esters developed by Donald S. Matteson was not only used in a variety of pharmaceutically and biologically relevant compounds [85,86,87,88,89,90], such as terpenes [91], carbohydrates [22], alkaloids [92] and pheromones [35,93,94,95,96], but also for the construction of a wide range of marine natural products.

### 3.1. Dictyopterene A

**Scheme 10:**
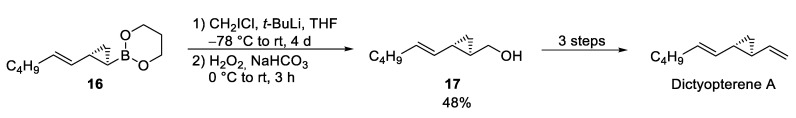
Synthesis of dictyopterene A according to Pietruszka et al. [98].

Dictyopterene A was isolated by Moore et al. as the main component of an odoriferous oil from seagrass of the species *Dictyopteris* [97]. It belongs to a group of sexual attractants that are found in various brown algae (*Phaeophyceae*). The various dictyopterene derivatives differ primarily in the position and in the number of unsaturations of the two substituents on the cyclopropane ring. In their synthesis of dictyopterene A, Pietruszka et al. used a Matteson homologation towards the end of their synthesis to obtain the primary alcohol **16** from cyclopropylboronic ester **16** by elongation with LiCH_2_Cl and subsequent oxidation (Scheme 10). Alcohol **17** was finally converted into the natural product by Dess–Martin oxidation and Wittig reaction [98].

### 3.2. Awajanomycin

Awajanomycin was extracted in 2006 from a marine fungus of the species *Acremonium* sp. AWA16-1. The compound shows moderate cytotoxicity in the mid-μ-molar range. Most conspicuous is its bicyclic structure formed by a lactone and a lactam ring [99].

**Scheme 11:**
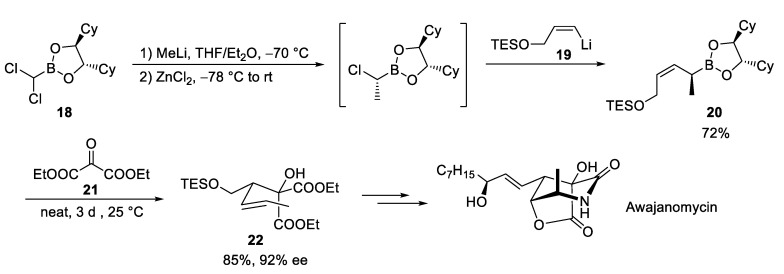
Synthesis of awajanomycin according to Koert et al. [100].

To set up the (*Z*)-configured allyl boronic ester **20**, Koert and coworkers reacted the dichloroboronic ester **18** first with MeLi and then with the (*Z*)-vinyllithium derivative **19**, whereby the desired allyl boronic ester **20** was obtained with good yield and selectivity (Scheme 11). Subsequent allylation of the tricarbonyl compound **21** yielded highly stereoselective compound **22**, which could be further converted into awajanomycin [100].

### 3.3. Danicalipin A

**Scheme 12:**
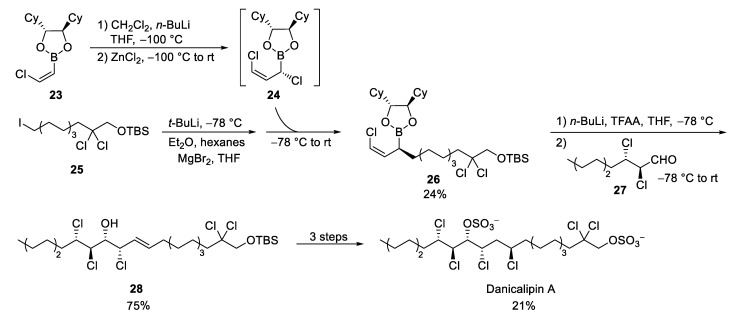
Synthesis of danicalipin A according to Burns et al. [102].

Danicalipin A belongs to a group of polychlorinated natural products called chlorosulfolipids. Similar to phospholipids, sulfolipids consist of a non-polar lipophilic and a polar hydrophilic part. They are mainly found in membranes of microalgae, e.g., danicalipin A is the main component of the cell membrane of *Ochromonas danica* [101]. Sulfolipids are usually cytotoxic and often responsible for seafood poisoning. Above all, the unusual structure has aroused the interest of synthetic chemists. A key step in the synthesis of the Burns group [102] was the extension of the chlorinated vinyl boronic ester **23** to the corresponding α-chloroboronic ester **24**, which was immediately further reacted to avoid decomposition (Scheme 12). For the incorporation of the first dichlorinated side chain, the primary iodide **25** was first lithiated with BuLi and then converted into the Grignard reagent before it was reacted with **24**. Transmetalation was necessary because the lithium compound proved to be unstable and some lithium-halogen exchange with the two chlorine atoms of the side chain was observed. The second half of the molecule was also introduced in a highly stereoselective manner by reacting **26** (>99%ee) with chlorinated aldehyde **27**. For this purpose, **26** was first converted into the borinate using *n*-BuLi and trifluoroacetanhydride [103,104], which yielded significantly better yields in the subsequent allylation than **26**, so that **28** could be obtained in gram scale. Danicalipin A was finally obtained in a few further steps.

### 3.4. Motuporin

Motuporin was isolated by Andersen and coworkers from the sponge *Theonella swinhoi Gray*, which was found around Papua New Guinea [105]. Motuporin belongs to the group of nodularins [106], very strong inhibitors of protein phosphatase 1 (PP1), and motuporin is one of the most active representatives. PP1 is inhibited at subnanomolar concentrations. In addition, motuporin also shows high cytotoxicity against various cancer cell lines. The most striking structural feature is the double unsaturated β-amino acid (2*S*,3*S*,8*S*,9*S*)-3-amino-9-methoxy-2,6,8-trimethyl-10-phenyl-4,6-decadienoic acid (ADDA), which is also found in other natural products such as microcystin [107]. Another unusual amino acid is (2*R*,3*S*)-2-methylaspartic acid. Both building blocks could be obtained by Matteson homologation via a common precursor.

To introduce *N*-functionalities either LiHMDS [108,109] or azide [110] can be used as nucleophiles. Ammonia and primary amines, on the other hand, are not suitable because the α-aminoboronic esters obtained are rather labile and decompose rapidly [111]. In contrast, reactions of α-chloroboronic acid esters with LHMDS yield stable silylated α-aminoboronic esters, which can be desilylated and acylated in a one-pot reaction. Further homologations of silylated α-aminoboronic esters are possible, but are usually slow and normally provide low yields [112,113]. In contrast, azides, generated from sodium azide under two-phase conditions in the presence of a phase transfer catalyst [23], are often tolerated in subsequent Matteson homologations.

**Scheme 13:**
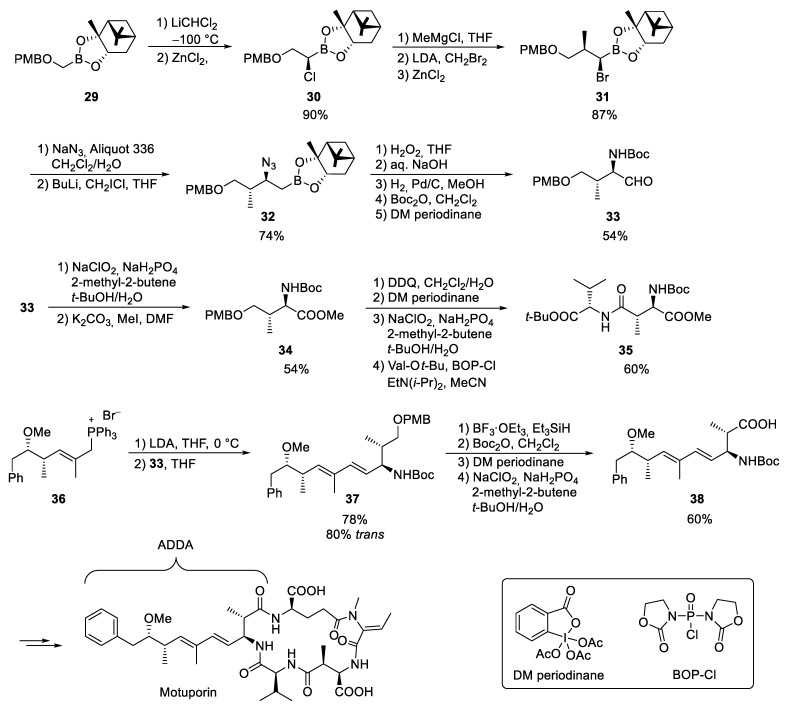
Synthesis of motuporin according to Armstrong et al. [114].

Bauer and Armstrong used this protocol to synthesize the unusual amino acids of motuporin (Scheme 13) [114]. Starting from the PMB-protected boronic ester **29**, the α-chloroboronic ester **30** was formed under standard conditions, which was further reacted with MeMgCl to form the α-methylated boronic ester **31**. Due to the relatively low nucleophilicity of the azide, the slightly more reactive α-bromoboronic ester was prepared for further elongation, which was reacted under Matteson’s phase transfer conditions. This was the only step in which some minor epimerization was observed [115]. The subsequent chain extension was carried out with LiCH_2_Cl to **32** before the boronic ester was finally oxidized to alcohol. The order in which the reaction components were added was of decisive importance. The hydrogen peroxide had to be added prior to the sodium hydroxide solution to obtain a reasonable yield. In reversed order, predominantly alkene was obtained, probably by elimination of the β-azidoboronate complex. After catalytic hydrogenation and Boc-protection of the free amine, the desired aldehyde **33** was obtained via Dess–Martin oxidation [116]. Aldehyde **33** was the common starting material for the synthesis of both amino acids.

For the synthesis of methyl aspartate, the aldehyde **33** was oxidized to carboxylic acid which was protected as methyl ester **34**. Subsequently, the PMB ether was cleaved with 2,3-dichloro-5,6-dicyano-1,4-benzoquinone (DDQ) [117] and the free alcohol was subjected to a Pinnick oxidation [118]. The free acid was coupled with *t*-butyl valinate to **35**. After cleavage of the Boc protecting group, the dipeptide was incorporated into the linear peptide chain.

To build up the ADDA, aldehyde **33** was subjected to a Wittig reaction with **36**, which gave the best results using LDA as a base. However, attempts to cleave the primary PMB ether of **37** with DDQ did not lead to the desired product, but to a cleavage of the *N*-Boc protecting group and oxidation of the amine to a ketone.

Therefore, the PMB ether was cleaved simultaneously with the Boc protecting group under acidic conditions and the free amine was protected again. Subsequent Dess–Martin (DM) and Pinnick oxidation yielded the desired unsaturated amino acid **38**, which was incorporated into motuporin.

### 3.5. Callipeltin A

The callipeltines also belong to a group of marine cyclopeptides with unusual amino acid building blocks [119,120,121,122]. Callipeltin A, the largest representative of the family, was isolated in 1996 by Zampella and colleagues from the marine sponge *Callipelta* sp. and shows interesting antiviral, antifungal and cytotoxic properties [10]. In addition to the amino acids (2*R*,3*R*)-β-methoxy tyrosine, (2*R*,3*R*,4*S*)-4-amino-7-guanidino-2,3-dihydroxy-heptanoic acid (AGDHA), (2*S*,3*S*,4*R*)-dimethyl-glutamine (diMeGln) and (*D*)-*allo*-threonine, the natural product also contains a *N*-terminal polyketide fragment (2*R*,3*R*,4*R*) 3-hydroxy-2,4,6-trimethylheptanoic acid (TMEHA). The latter three building blocks were obtained by Horn and Kazmaier via Matteson homologation [123,124].

**Scheme 14:**
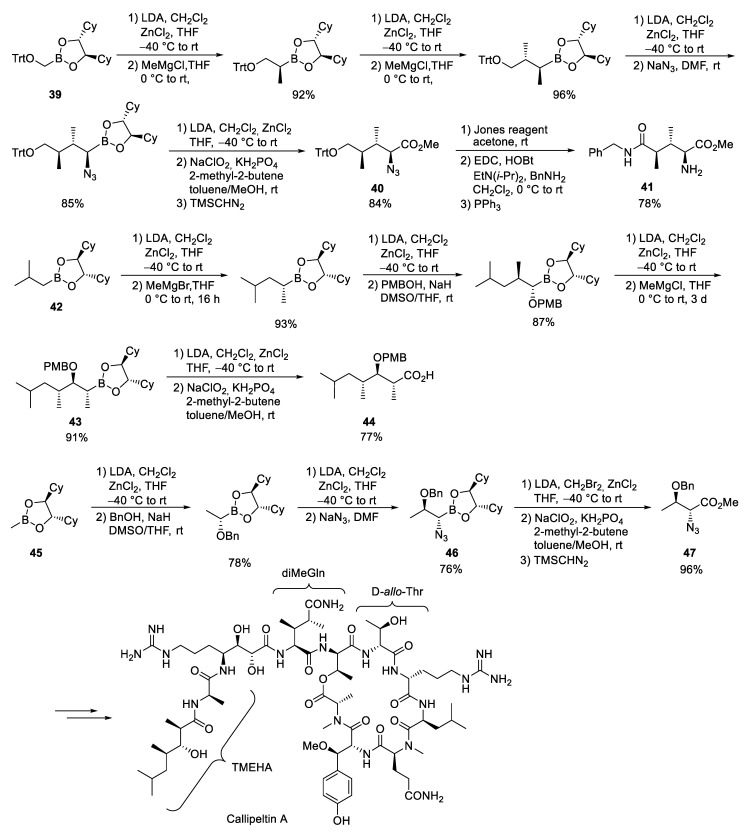
Synthesis of callipeltin A according to Horn and Kazmaier [123,124].

The *N*-benzyl-protected DiMeGln was obtained starting from boronic ester **39**, which was extended in four successive homologation steps to **40** (Scheme 14). The two methyl groups were introduced by the reaction of the corresponding α-chloroboronic esters with MeMgCl. The azide was generated here not under phase transfer conditions but using sodium azide in DMF [125,126]. Final homologation and direct oxidation of the α-chloroboronic esters under Pinnick conditions yielded the carboxylic acid which was subjected to esterification. However, the trityl residue could not be removed by catalytic hydrogenation, as spontaneous lactonization was observed. In contrast, direct Jones oxidation delivered the desired acid which was reacted with benzylamine to amide **41** in overall good yield.

The β-hydroxy acid building block TMEHA was obtained analogously from isobutyl boronic ester **42**. The protected OH functionality was introduced by using sodium *p*-methoxybenzylate as a nucleophile. The subsequent implementation with MeMgBr was extremely slow, even after 14 days no complete conversion could be achieved. In contrast, the reaction with the more reactive MeMgCl was complete after 3 days. The final homologation of **43** and Pinnick oxidation gave rise to the desired acid **44**.

The (D)-*allo*-threonine, which is commercially available but rather expensive, was obtained analogously from methylboronic ester **45**. Interestingly, the elongation of the azido boronic ester **46** had to be carried out with deprotonated dibromomethane to suppress side reactions. Subsequent oxidation yielded the desired acid in almost quantitative yields. After esterification, the azide **47** was reduced and the free amine was Alloc-protected before the methyl ester was saponified. Classical peptide coupling chemistry was used to obtain protected callipeltin A.

### 3.6. Tautomycin

**Figure 2:**
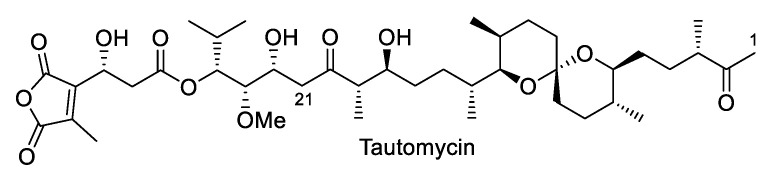
Tautomycin.

Tautomycin, which is found in various shellfish, was isolated by Isono et al. from the fermentation broth of *Streptomyces spiroverticillatus* [127]. It is a selective inhibitor of the serine/threonine phosphatases PP1 and PP2a but shows no structural similarity to motuporin, which shows a similar biological effect. Tautomycin contains an interesting spiroketal unit and a terminal unsaturated anhydride (Figure 2).

In connection with the synthesis of various PP1 inhibitors, Maurer and Armstrong also developed a synthesis for the right-hand C1–C21 fragment of tautomycin, based on the Matteson homologation [128]. This fragment was then to be coupled to the left half of the molecule via an aldol reaction.

**Scheme 15:**
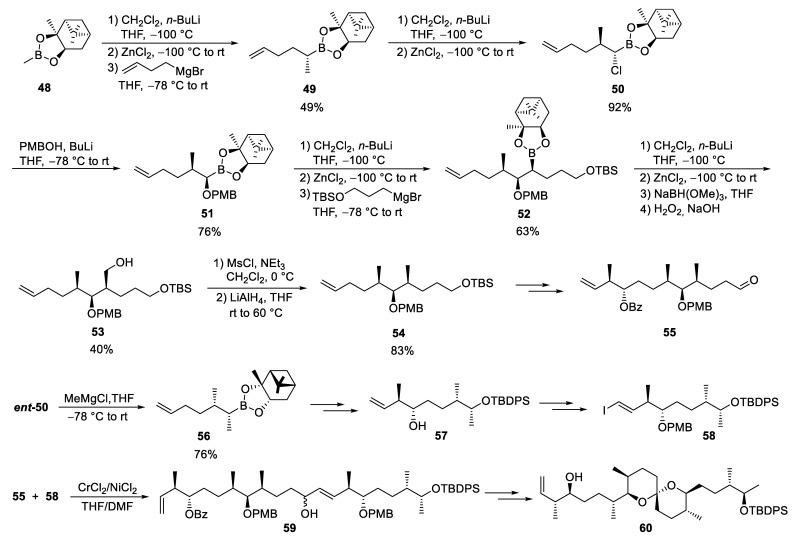
Synthesis of tautomycin according to Maurer and Armstrong [128].

The right half of the molecule was built up from two fragments, both obtained via Matteson homologations, which were then linked via a Ni/Cr coupling (Scheme 15). Starting the synthesis chiral boronic acid ester **48** was extended to the corresponding α-chloroboronic ester (61%) and further reacted with 3-butenylmagnesium bromide to **49**. The Grignard reagent was slowly added until the entire α-chloroboronic ester was consumed. The next extension to the α-chloroboronic ester **50** was unproblematic (92%), but the addition of lithium *p*-methoxybenzyl alcoholate had to be carefully controlled. The reaction was aborted after the chloroboronic ester was consumed. Longer reaction times caused a drop in the yield of **51**. In the next extension step, a TBS-protected Grignard reagent proved superior to the THP-protected derivative, which was prone to the formation of by-products. Further elongation of the boronic acid ester **52** and oxidation provided the primary alcohol **53**, which was reduced with LiAlH_4_ after activation as a mesylate. Compound **54** was then converted into aldehyde **55** via further standard operations, which represents the C-10 to C-21 fragment of the desired building block.

The second part of the fragment started from the enantiomeric boronic ester *ent*-**48**, which was converted into the enantiomeric α-chloroboronic ester *ent*-**50** as described. Reaction with methylmagnesium chloride yielded boronic ester **56**. Oxidation and protection of the secondary alcohol, ozonolysis followed by crotyl boration yielded homoallyl alcohol **57**, which gave rise to the desired vinyl iodide **58** after PMB-protection, ozonolysis and subsequent Takai reaction [129]. Kishi–Nozaki coupling [130,131] of **55** and **58** using NiCl_2_-doped CrCl_2_ yielded the allyl alcohol **59**, which could ultimately be converted into the desired fragment **60**.

### 3.7. Emericellamide A

**Scheme 16:**
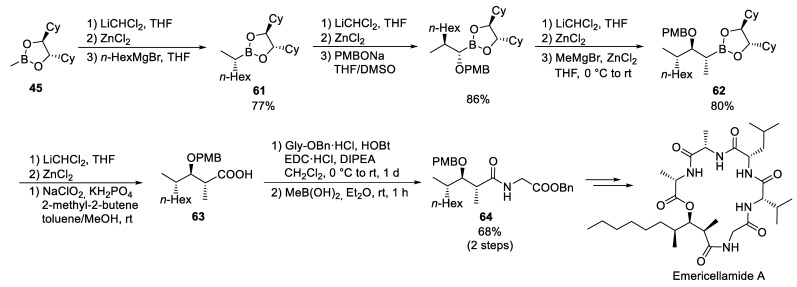
Synthesis of emericellamide A according to Priester and Kazmaier [132].

In 2007, Fenical and co-workers described the isolation of the two cyclic lipodepsipeptides emericellamide A and B from the marine fungus *Emericella* sp. Both compounds show activity against methicillin-resistant *Staphylococcus aureus* (MRSA) strains in the low μ-molar range, with emericellamide A being the slightly more active compound (3.8 μM (A) vs. 6.0 μM (B)). The moderate cytotoxicity of both compounds is striking. For the synthesis of emericellamide A and the formation of β-hydroxycarboxylic acid **63**, Priester and Kazmaier started their synthesis from the well-known methylboronic acid ester **45** [30,37], which was reacted with hexylmagnesium bromide under standard conditions to the homologated boronic acid ester **61** (Scheme 16) [132]. The next homologation with *p*-methoxybenzylate was performed in the presence of DMSO to accelerate the 1,2-migration of the alkoxy nucleophile [22]. However, the subsequent introduction of a methyl group was very slow under the usual conditions. However, the reaction could be accelerated by isolating the α-chloroboronic acid ester and reacting with the methyl Grignard reagent. Further Matteson homologation of **62** and Pinnick oxidation yielded carboxylic acid **63**. Unfortunately, separation of the product from the auxiliary was not possible at this time, so **63** was directly linked to glycine benzyl ester (**64**). After esterification of the chiral diol with methylboronic acid (under regeneration of **45**) and PMB deprotection, the resulting alcohol could be incorporated into the peptide.

### 3.8. Lagunamides A

Lagunamides A and B, isolated from the marine cyanobacterium *Lyngbya majuscula* [8] belong to an interesting class of structurally closely related marine cyclodepsipeptides, such as kulokekahilide-2,2 [133,134,135] odoamide1 [136] and aurilide [137,138,139]. Both lagunamides show interesting biological properties, e.g., nanomolar cytotoxicity against various tumor cell lines as well as interesting anti-malarial activity. Lagunamide A stimulates caspase- and mitochondria-mediated apoptosis in tumor cells [140]; however, the exact target was not known until now. Very recently, based on thermal protein profiling (TPP) experiments, Sieber, Zahler and coworkers could identify EYA3 as a stabilized protein in cells upon lagunamide A treatment. Furthermore, they could show that lagunamide A sensitized tumor cells to treatment with doxorubicin, highlighting a putative therapeutic strategy [141].

**Scheme 17:**
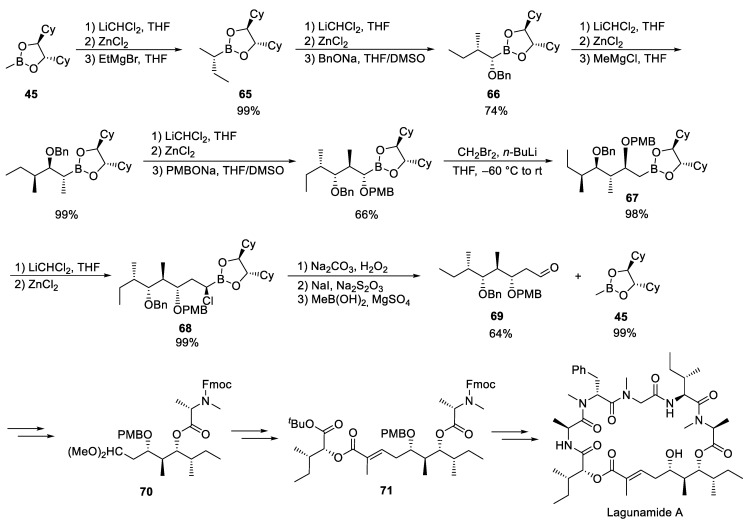
Synthesis of lagunamide A according to Gorges and Kazmaier [24].

In their synthesis of lagunamide A, Gorges and Kazmaier also started with methylboronic acid ester **45**, which was subjected to Matteson homologation with ethyl Grignard reagent, whereby boronic acid ester **65** was formed diastereomerically pure in almost quantitative yield (Scheme 17) [24]. In the next step, the raw chloroboronic ester formed was reacted directly with benzyl alcoholate providing ester **66**. The next homologation step with MeMgCl went smoothly but required a rather long reaction time of 14 days. The formation of the last stereogenic center was achieved by a further homologation step using *p*-methoxybenzylate. For the introduction of the CH_2_ group, lithiated bromomethane was generated from dibromomethane with BuLi [40]. The final homologation from **67** to **68** was unproblematic, and the aldehyde **69** was finally obtained by oxidation of the α-chloroboronic acid ester **68**. To improve purification, the raw mixture was treated with methylboronic acid, which allowed the formation of **69** as well as the near-quantitative recovery of the initial boron ester **45**.

To complete the polyketide unit, the aldehyde **69** should be converted to the α,β-unsaturated ester in a Horner–Wadsworth–Emmons reaction. However, since the benzyl protection group could not be cleaved selectively in the presence of the α,β-unsaturated ester in the further course of synthesis, the aldehyde was first protected and the polyketide fragment was linked to *N*-methylalanine (N-Me-Ala) after cleavage of the benzyl ether (**70**). After acetal cleavage, the subsequent Horner–Wadsworth–Emmons reaction to **71** gave the best results when the lithium salt of hexafluoroisopropanol (HFIP) was used as a base. The further construction of the lagunamide A did not cause any further problems.

**Scheme 18:**
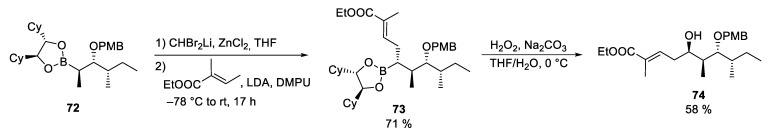
Synthesis of polyketide fragment **74** according to Andler and Kazmaier [143].

It was known from SAR studies with the structurally closely related aurimide and analogs that the hydroxyl group of the polyketide has only a minor influence on biological activity and can be removed or acylated without much loss of activity [142]. Therefore, a shortened synthesis route was developed that allowed access to a diastereomeric polyketide part, based on the use of ester dienolates as nucleophiles. Matteson had reported that deprotonated *tert*-butyl esters are suitable as nucleophiles [23,24], and Andler and Kazmaier have now extended this concept to deprotonated α,β-unsaturated esters [143]. In this case, the OH group is not generated by S_N_2 reaction, but by oxidation of the boric acid ester and therefore has the opposite configuration. Starting from building block **72**, which already contains the first three stereogenic centers, homologation with CHBr_2_Li and the subsequent reaction with the dienolate of the ethyl tiglate yielded the extended boronic ester **73**, which was then oxidized to the desired polyketide fragment **74** (Scheme 18). However, incorporation into the isomeric lagunamide showed that in this case, an inversion of the stereocenter led to a significant drop in biological activity.

### 3.9. Apratoxin A and B

**Figure 3:**
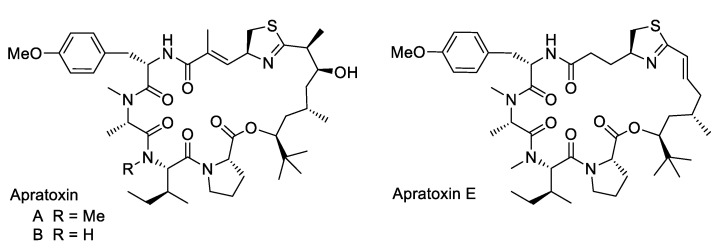
Selected apratoxins.

Apratoxins are another interesting class of cyclodepsipeptides produced by marine cyanobacteria [144,145,146,147]. The first representative of this group, apratoxin A, isolated from the cyanobacterium *Lyngbya majuscula*, was described in 2001 by Moore and Paul et al. [9]. Among other activities, it showed high cytotoxicity against a number of tumor cell lines in the subnanomolar range [148,149,150]. In the following years, further apratoxins were isolated [146,151], some of which are shown in Figure 3. The apratoxins form a 25-membered ring consisting of a pentapeptide and a rather unusual polyketide fragment, which in most cases contains a terminal *t*-butyl group. They differ primarily in the methylation pattern and in the structure of the polyketide fragment, slight variations are also found in the peptide part.

**Scheme 19:**
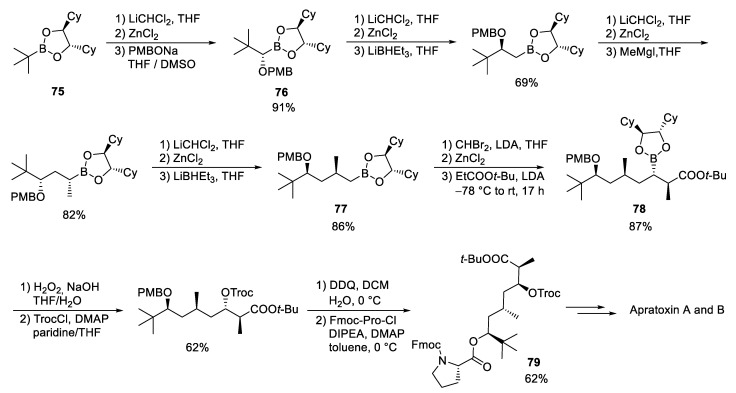
Syntheses of apratoxins A and B according to Andler and Kazmaier [152].

Andler and Kazmaier developed a Matteson homologation-based synthesis of apratoxin A and B starting from chiral boronic ester **75** (Scheme 19) [152]. Regardless of the steric hindrance by the *t*-butyl group, the conversion to the corresponding α-chloroboronic ester was successful, which could be reacted with an excess of alcoholate to **76**. Three further homologation steps resulted in boronic ester **77**, which was stereoselectively reacted with the Li-enolate of *t*-butylpropionate (*d.r.* 9:1) to **78** in a final extension step [8,24]. The secondary alcohol formed by oxidation was temporarily Troc-protected before the PMB protection group was removed.

The coupling of the alcohol formed with the acid chloride of Fmoc-proline yielded the ester **79** in excellent yield and without epimerization of the α stereocenter at the proline. The acidic cleavage of the *t*-butyl ester resulted in the free carboxylic acid, which could finally be incorporated into the desired apratoxins. During that, partial epimerization of the stereocenter adjacent to the thiazoline unit was observed, but the isomers could be separated by preparative HPLC.

### 3.10. Doliculide

The isolation of (–)doliculide from the Japanese seahare, *Dolabella auricularia*, was first reported by Yamada et al. almost 30 years ago, together with the pronounced cytotoxicity of this compound against HeLa-S3 cells (IC_50_ = 1 ng/mL) [153]. The nudibranch itself does not necessarily have to be the producer of this cyclic depsipeptide, as it has been shown that other metabolites isolated from *Dolabella auricularia* often come from cyanobacteria and are therefore of dietary origin [154]. Doliculide is a potent actin binder that initiates actin aggregation, resulting in inhibition of proliferation and apoptosis [155,156,157,158,159]. It was shown that doliculide stabilizes F-actin in a similar way to structurally related natural products such as jaspamid or miuraenamide [160,161,162]. Subtoxic doses of doliculide result in a transient change in reversible cytoskeletal dynamics and induction of premature senescence in p53 wild-type cells [163].

**Scheme 20:**
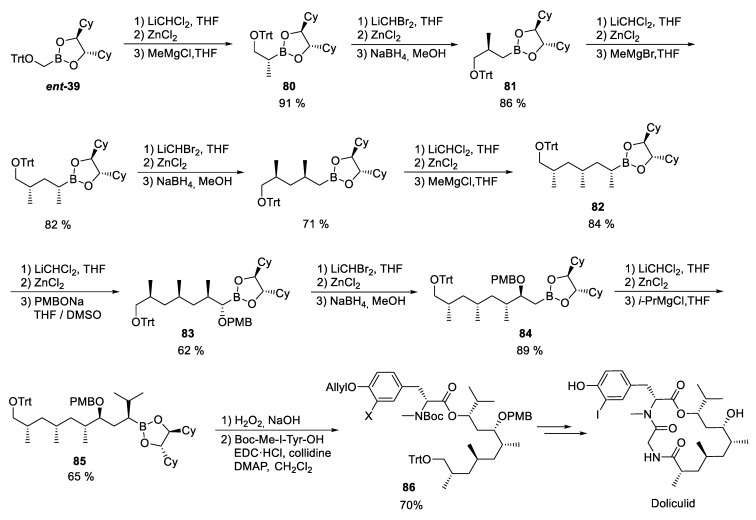
Synthesis of doliculide according to Kazmaier et al. [44].

Doliculide consists of a halogenated dipeptide segment and a polyketide unit with five stereogenic centers that can be built up by Matteson homologation (Scheme 20). In order to be able to specifically vary the C-terminal end of the polyketide fragment at a late stage of the synthesis, Kazmaier and coworkers chose the sequential construction of the polyketide in the opposite direction as the natural biosynthesis [44].

Starting from the trityl-protected boronic ester *ent***-39** [164], the reaction with deprotonated methylene chloride and subsequently with the Grignard reagent in a one-pot reaction yielded the extended boronic ester **80**. The next extension step was carried out with lithiated dibromomethane and the α-bromoboronic ester was reduced with NaBH_4_ to **81**. The repetition of these steps led to the boronic acid ester **82**, which was homologated with *p*-methoxybenzylate to **83**. The next CH_2_ insertion to **84** went well, while the introduction of the final isopropyl group required some optimizations. In this case, the direct reaction of the α-chloroboronic ester resulted in a mixture of several products. Better results were obtained when the α-chloroboronic ester was isolated before further conversion.

The final oxidation of **85** led to the desired protected polyketide precursor which was coupled with *O*-allylated halogenated tyrosine to **86**. Further standard operations gave rise to doliculide. Boronic ester **84** was reacted in an analogous way also with other Grignard reagents (methyl, allyl, progargyl), which allowed the construction of derivatives that could be further modified, e.g., by thiol-ene-click chemistry or [3+2]-cycloadditions [165].

## 4. Syntheses of Marine Natural Products Using the Lithiation–Borylation Protocol

The lithiation–borylation homologation strategy is based on Hoppe’s chiral deprotonated carbamates [50,51,52,53,54,55] or Beak’ aryl esters [56] and has been used by Aggarwal and others in various ways for natural product syntheses [59,166], including a range of marine natural products.

### 4.1. Solandelactone E and F

Soledanolactones, a group of marine oxylipids, were first isolated from *Solanderia secunda* by Shin and coworkers [167]. As a striking structural feature, they contain an 8-membered lactone ring linked to a *trans*-configured cyclopropane. In soledanolactone E, this unit is linked to a double unsaturated fatty acid residue via an unsaturated *anti*-1,4-ene-diol, while in soledanolactone F this subunit has a *cis* configuration.

**Scheme 21:**
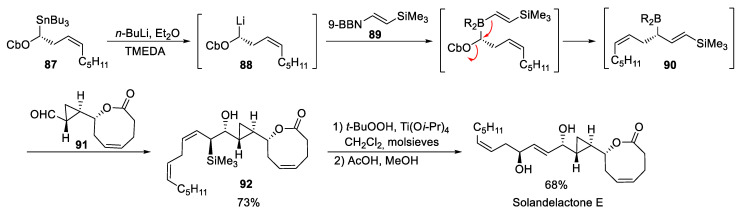
Synthesis of soledanolactone E according to Aggarwal et al. [169].

In 2010, Aggarwal and co-workers reported on an elegant method for the stereoselective synthesis of such unsaturated *anti*-1,4-diols based on the reaction of chiral lithium carbamates with β-silylvinylboranes [168]. Sparteine-complexed lithiated carbamates react with alkylboranes under inversion, while retention can be observed in the absence of sparteine or other sterically demanding diamine ligands. A 1,2-Shift of the vinyl residue generates chiral allylboranes which can be subjected to allylborations with aldehydes in a highly enantioselective fashion. Due to the steric demand of the bicyclononane scaffold on the boron, the allylic substituent preferentially occupies an axial position in the Zimmermann–Traxler transition state, which ultimately leads to the *anti*-configured product. This reaction sequence was used in the synthesis of soledanolactone A (Scheme 21) [169].

For this purpose, the chiral tin reagent **87** was transmetalated with *n*-BuLi (e.r. 10:1) giving rise to an “diamine free” lithiated carbamate **88** which was reacted with the silylated vinyl borane **89** to provide allylborane **90**. The addition of aldehyde **91** to the in situ formed **90** provided the desired allylsilane **92** initially in a rather low yield (15%). The problem resulted from the poor stirrability of the viscous reaction mixture. By adding TMEDA in the transmetalation step, this problem was solved and the yield could be increased to 73%. The diastereomers (i.e., 10:1) could be separated at this stage, whereby the lithium-boron exchange took place under complete retention even in the presence of TMEDA. Sharpless epoxidation of **92** allowed the selective oxidation of the homoallyl alcohol in the presence of the other two double bonds [170]. Acid-catalyzed ring-opening-elimination of the α-silylated epoxide yielded diastereomerically pure soledanolactone E.

**Scheme 22:**
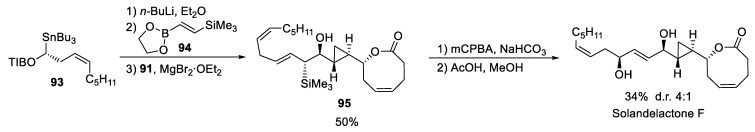
Synthesis of soledanolactone F according to Robinson and Aggarwal [171].

Robinson and Aggarwal also developed a slightly modified route that leads to *syn*-2-ene-1,4-diols, such as soledanolactone F [171]. In principle, if the (*E*)-configured allyl silane is preferentially formed in the allylation step, this should provide the *syn*-1,4-ene-diol under the same oxidation/elimination conditions. This could be achieved by using β-silylvinyl-boronic acid ethylene glycol ester. However, boronic esters enter into a 1,2-alkyl shift much more slowly than alkyboranes, and allylboration is usually also slower, but can often be accelerated by adding Lewis acids. For example, no vinyl migration was observed when homoallylcarbamate **87** was reacted with vinyl boronic ester **94**, but it occurred when Beak’s TIB ester **93** was used (Scheme 22) [56]. The reaction of the allylborane formed with **91** in the presence of MgBr_2_ yielded the desired (*E*)-allyl silane **95** in reasonable yield and selectivity. Epoxidation with *m*CPBA and acidic elimination in this case yielded soledanolactone F but only in moderate yield and selectivity.

### 4.2. Erogorgiaene

**Scheme 23:**
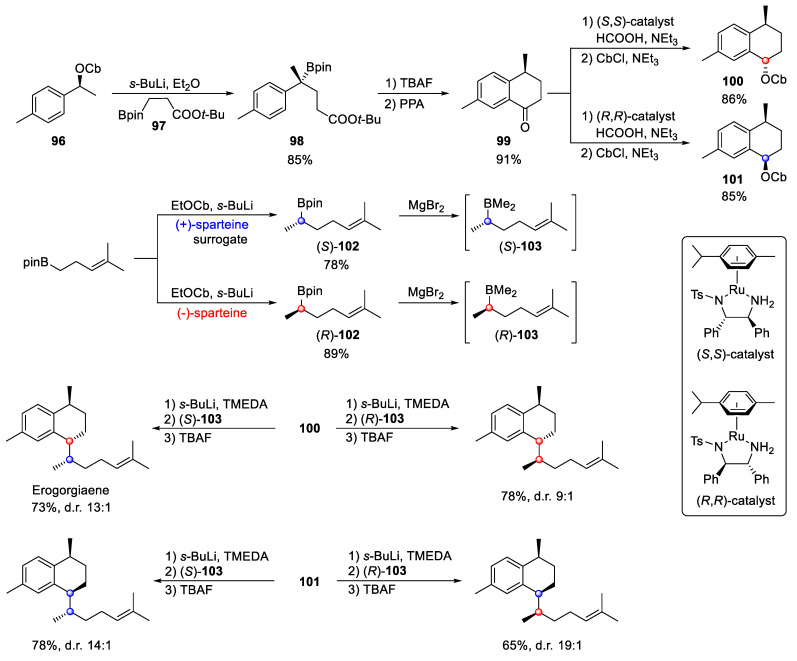
Synthesis of erogorgiaene and stereoisomers according to Aggarwal et al. [175].

Erogorgiaene is a marine diterpene natural product isolated from the Pacific horn coral *Pseudopterogorgia elisabethae* that shows promising activity against *Mycobacterium tuberculosis* H37Rov [172]. By applying the lithiation/borylation strategy in combination with protodeborations, the synthesis of four stereoisomers of the erogorgia was achieved. The starting point was the chiral benzyl carbamate **96**, which is easily accessible from *p*-methylacetophenone by Noyori reduction [173]. Reaction with *s*-BuLi and addition of boronic ester **97** yielded tertiary boronic ester **98**, which then protodeborates with TBAF [174] with retention of the configuration (Scheme 23) [175]. Treatment with polyphosphoric acid (PPA) yielded ketone **99**, which was likewise reduced with either the (*S*,*S*) or (*R*,*R*)-Noyori catalyst to obtain the corresponding *trans* and *cis* alcohols enantiomerically pure, in order to subsequently convert them into the desired carbamates **100** and **101**. Such benzylic carbamates can be converted into the corresponding boronates either under retention or inversion, depending on whether alkylborans or boronic esters are used [63]. Model reactions have shown that alkyl dimethylboranes yield the best selectivities with such carbamates, with sterically demanding and seconded alkyl groups migrating preferentially. Therefore, for the introduction of the unsaturated side chain, the chiral boronic pinacol esters **102**, which were accessible via the sparteine route, were converted with MeMgBr into the corresponding dimethylboranes **103**, which were reacted directly with the carbamates **100** and **101**. Final protodeborations with TBAF yielded the desired erogorgiaene and its diastereomers with *d.r.* ≥ 9:1.

### 4.3. Sporochnol A

**Scheme 24:**
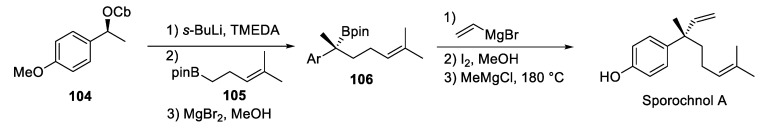
Synthesis of sporochnol A according to Aggarwal et al. [180].

Sporochnol A, isolated from the Caribbean marine alga *Sporochnus bolleanus*, has been shown to exhibit significant feeding deterrence towards herbivorous fish [176]. It can easily be obtained similar to Erogorgiaene from chiral benzyl carbamate **104** (Scheme 24). Reaction with the unsaturated boronic ester **105** yielded the tertiary boronic ester **106**, which was subjected to a Zweifel-olefination [177,178,179]. The final step was the cleavage of the methyl ether [180]. In a similar way, a number of sesquiterpenes of the Bisabolane family have been obtained [181].

### 4.4. Aplysin

**Scheme 25:**
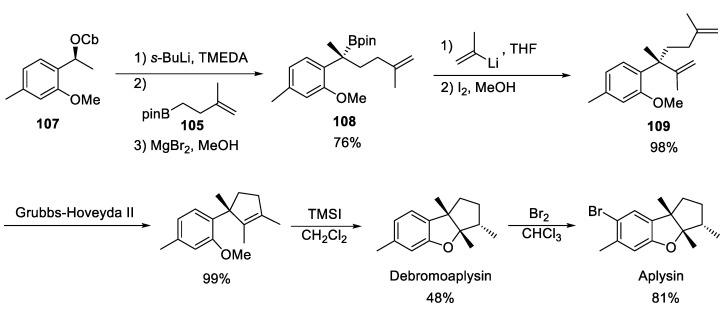
Synthesis of debromoaplysin and aplysin according to Aggarwal et al. [184].

Marine mollusks of the *Aplysia* species are known to feed almost exclusively on *Laurencia* algae and store their metabolites with anti-feeding properties to protect themselves from predators [182]. A wide range of sesquiterpenoid natural products have been isolated from these organisms over the last 60 years. Typical representatives are e.g., aplysin and debromoaplysine [183], which are easily accessible via Aggarwal’s lithiation-borylation route (Scheme 25) [184].

Again, the key steps were the reaction of a lithiated secondary benzylic carbamate **107** to obtain a tertiary boronic ester **108**, followed by propenylation in which the quaternary stereocenter of **109** was constructed with perfect enantioselectivity. The subsequent ring-closing metathesis, followed by deprotection and in situ cyclization, gave rise to debromoaplysin with good diastereoselectivity. Subsequent bromination provided access to aplysin.

### 4.5. Filiformin

**Scheme 26:**
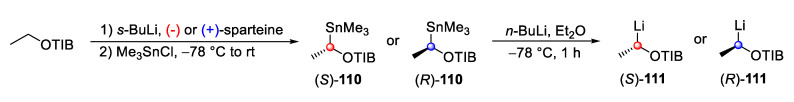
Generation of “diamine-free” chiral lithiated primary carbamates **111**.

Filiformin is a sesquiterpene natural compound, isolated from the red seaweed *Laurencia filiformis*, that contains quaternary stereogenic centers [185]. In the synthesis, both a chiral lithiated secondary benzylic and a primary carbamate **111** were used. The latter was generated by transmetalation of the chiral tin compound **110**, which allowed a “diamine-free” reaction (Scheme 26).

**Scheme 27:**
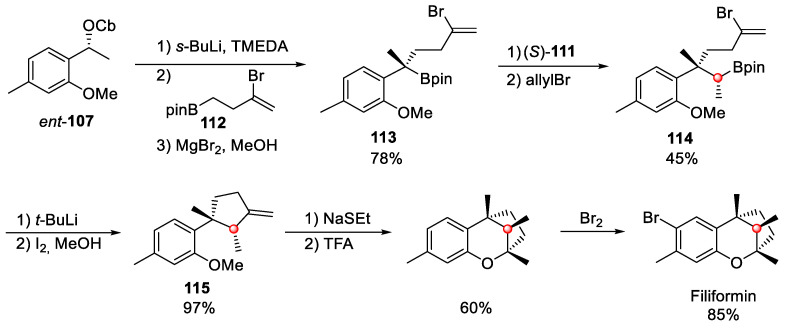
Synthesis of filiformin according to Aggarwal et al. [186].

The starting point of the synthesis was the chiral carbamate *ent*-**107**, which, after lithiation, was reacted with the halogenated unsaturated boronic ester **112** to **113** (Scheme 27) [186]. A second homologation with freshly produced (*S*)-**111** delivered the extended boronic ester **114** with perfect enantio- and diastereoselectivity. The key to the success of this challenging homologation was the use of the diamine-free carbenoid **111** and the addition of allyl bromide to quench the benzylic carbanion formed during the 1,2 rearrangement. An intramolecular Zweifel-type olefination yielded methylene cyclopentane **115** in excellent yield. The cleavage of the aromatic methyl ether, acid-catalyzed cyclization and bromination completed the synthesis of the natural product.

### 4.6. Kalkitoxin

(+)-Kalkitoxin is a neurotoxic lipopeptide isolated from the marine cyanobacterium *Lyngbya majuscula* [187]. The compound showed strong neurotoxicity [188] by blocking the voltage-sensitive Na^+^ channel [189]. In this case, only freshly generated lithiated benzoate esters (*S*)-**111** and (*R*)-**111** (Scheme 26), were used to build up the three stereogenic centers of the central polymethylated ω-aminocarboxylic acid [190]. For the introduction of the CH_2_ units, LiCH_2_Cl (**116**) was freshly prepared using the standard Matteson protocol [38,39].

**Scheme 28:**
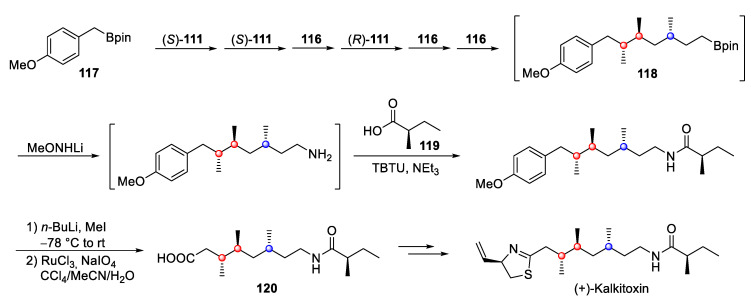
Synthesis of kalkitoxin according to Aggarwal et al. [192,193].

The synthesis of kalkitoxin started with achiral *p*-methoxybenzyl boronic ester **117** [191], which was successively converted twice with (*S*)-**111**, once with **116**, once with (*R*)-**111** and then twice more with **116** to form the elongated boronic ester **118** (Scheme 28) [192,193]. To introduce the terminal amino group, lithiated *O*-methylhydroxylamine was added [194] and the resulting primary amino group was acylated with **119** and subsequently *N*-methylated. Oxidative cleavage of the electron-rich aromatic ring led to carboxylic acid **120**, which was converted into kalkitoxin.

### 4.7. Clavosolide A

The clavosolides A–D were isolated from extracts of the sea sponge *Myriastra clavosa,* which was found near the Philippines [195,196]. Crude extracts of *Myriastra clavosa* showed promising cytotoxic and antiproliferative effects in antitumor screenings. However, a detailed investigation of the biological properties was not possible due to the limited quantities available. However, the substance class is not only interesting for synthetic chemists because of its biological activities, but also because of its complex structure. Clavosolide A is a symmetrical dimeric macrolide with highly substituted cyclopropyl, tetrahydropyranyl and glycosidic ring systems.

**Scheme 29:**
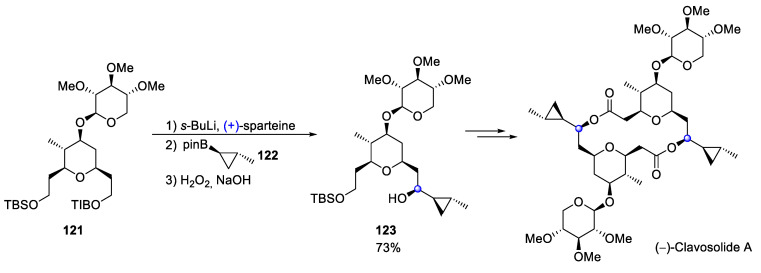
Synthesis of clavosolide A according to Aggarwal et al. [197].

As part of their synthesis of clavosolide A, Aggarwal and co-workers wanted to show that their lithiation-borylation method can also be used for such complex structures as **121** and used it as a late-stage coupling to perform the last stereoselective C-C coupling (Scheme 29) [197]. The TIB ester proved to be superior as a coordinating group to the initially used Cb-carbamate, as the latter provided only very moderate and varying yields (23–48%) due to competing deprotonation at the glycoside ring. This problem could be circumvented by using the more acidifying TIB-group, which increased the yield in the reaction of **121** with cyclopropane boronic ester **122** to 73%. Direct oxidation of the generated boronic ester yielded the alcohol **123**, which could be converted into clavosolide A in three further standard reactions.

### 4.8. Baulamycin A and B

Analysis of extracts of *Streptomyces tempisquensis*, marine bacteria found in the coastal region of Costa Rica led to the isolation of the novel antibiotics baulamycins A and B. These two polyketides are active against the superbug methicillin-resistant *Staphylococcus aureus* (MRSA) and *Bacillus anthracis* [198] as well as several other important bacterial pathogens. Liquid culture studies demonstrated the capacity of these natural products to penetrate bacterial barriers and inhibit the growth of both Gram-positive and Gram-negative species. However, extensive biological studies could not be carried out due to the small amount of material available, and only the smallest amounts of isolated natural products were available for NMR spectroscopic structure determination.

**Scheme 30:**
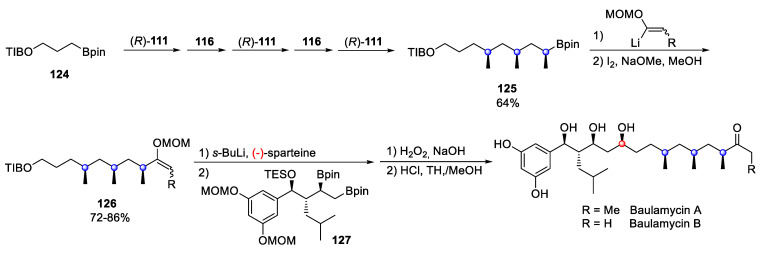
Syntheses of baulamycin A and B according to Aggarwal et al. [199].

The substitution pattern of the polyketide part is predestined for the application of the assembly line protocol, especially since it allows arbitrary inversion of individual stereocenters. Aggarwal and colleagues were thus able to correct the originally postulated structure by synthesizing various stereoisomers [199]. Their synthesis was based on the linking of two fragments at the central hydroxyl group based on the lithiation-borylation strategy (Scheme 30). The right half of the molecule was converted from boronic ester **124** via assembly-line synthesis into the boronic ester **125**, which was then subjected to a Zweifel olefination. Using a lithiated enol ether in this step yielded **126** as masked ketone. Deprotonation of **126** in the presence of (−)-sparteine followed by a regioselective homologation of the primary boronic ester of **127** resulted in the corresponding 1,3-bis(boric acid ester), which, after oxidation, gave rise to the desired diol in a highly stereoselective fashion. Acid hydrolysis of the protective groups led to the natural products.

### 4.9. Bastimolide B

**Figure 4:**
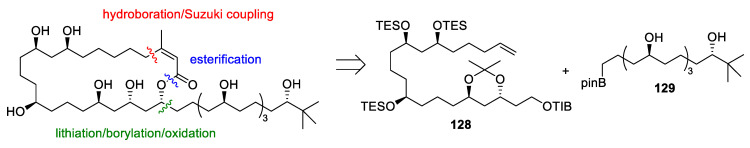
Retrosynthesis of bastimolide B.

Bastimolide B is a 24-membered polyhydroxylated macrolide isolated from marine cyanobacteria *Okeania hirsute* [200] and, like bastimolide A, has high antimalarial activity, also against multidrug-resistant strains of *Plasmodium falciparum* [201]. Structurally striking is the dense arrangement of hydroxylated stereogenic centers with a 1,5 relationship along a hydrocarbon chain (Figure 4). These 1,5-polyols represent a particularly challenging subject for synthesis, as the distant position of the stereocenters makes their stereoselective generation difficult [202]. To build up such structural motifs, Aggarwal and coworkers have extended their synthetic approach for 1,4-diols to the stereoselective assembly of 1,5 diols [203]. Retrosynthetically, bastimolide B can be split into two main fragments. One is the lactone ring (left half) and the other is the 1,5-polyhydroxylated side chain (right half). The carboxyl terminus of the lactone should be accessible from a terminal alkene via hydroboration/Suzuki coupling. This resulted in the main fragments **128** and **129** that had to be synthesized.

**Scheme 31:**
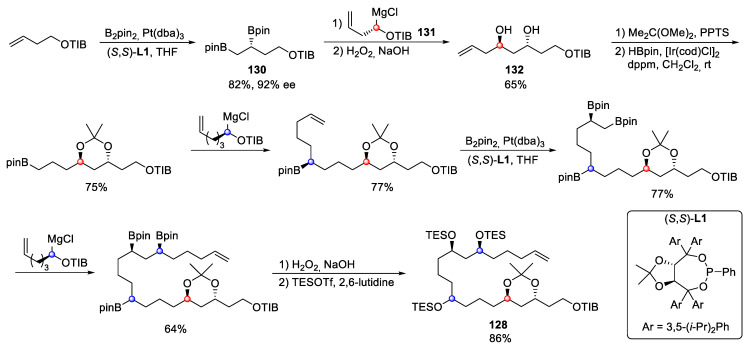
Syntheses of bastimolide fragment **128**.

The synthesis of fragment **128** started with bisboronic ester **130**, which was obtained from the corresponding homoallylic TIB ester by enantioselective Pt-catalyzed diboration (Scheme 31) [204,205]. Homologation of the primary boron ester with **131**, which was generated in situ from a stable sulfoxide [206,207], and subsequent oxidation yielded 1,3-diol **132**. Repeated enantioselective Pt-catalyzed diboration/homologation/oxidation and final silyl protection yielded the desired building block **128**.

**Scheme 32:**
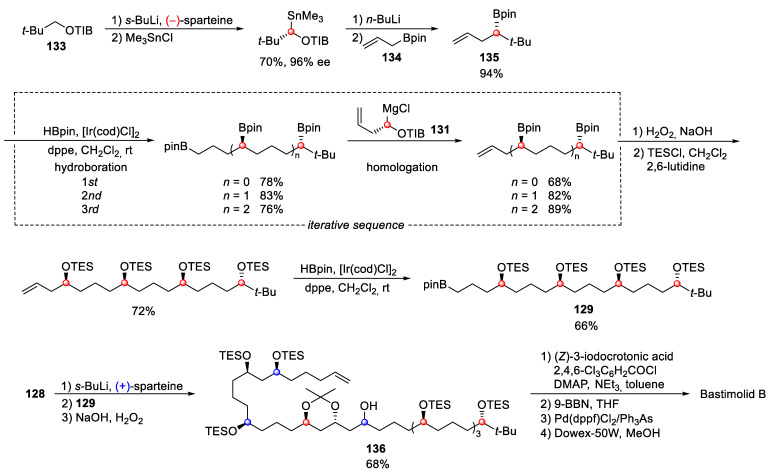
Syntheses of bastimolide B according to Aggarwal et al. [203].

The synthesis of fragment **129** started with the neopentyl TIP ester **133** (Scheme 32). Deprotonation with *s*-BuLi in the presence of (−)-sparteine and subsequent reaction with allyl boronate **134** yielded the desired product **135** with 96% ee, but only in 17% yield, probably due to the steric demand of the sparteine-lithium-carbenoid complex. Therefore, “diamine-free” conditions were used, in which the lithium carbenoid was first transmetalated to the corresponding tin compound and then regenerated on demand “sparteine-free”. Under these conditions, the yield of **135** could be increased to 94% (NMR). The crude product was subjected to iridium-catalyzed hydroboration [208,209,210], and subsequent reaction with magnesium carbenoid **131** yielded the diastereomerically pure 1,5-bisboronic ester. After repeating this sequence twice, the tetraboronic ester was obtained on a gram scale. Oxidation and silyl protection of the tetrol formed yielded the desired fragment **129** after final hydroboration.

To connect the two fragments, a slight excess (1.5 equiv.) of **128** was deprotonated in the presence of (+)-sparteine before **129** was added, which led to linear precursor **136** in excellent stereoselectivity after oxidation. Esterification of the secondary alcohol with (*Z*)-3-iodocrotonic acid, hydroboration of the terminal alkene, and intramolecular Suzuki coupling [211,212,213] provided the desired macrolactone, which was deprotected under acidic conditions to bastimolide B.

### 4.10. Rakicidin F

The rakicidines are a family of cyclic depsipeptides isolated from *Streptomyces* sp. and which partially inhibit the invasion of tumor cells [214]. Rakicidin F was extracted from the fermentation broth of the actinomycete strain *Streptomyces* sp. GKU 220, which was isolated from a sea sponge collected in the Andaman Sea near Thailand [215]. Rakicidin F showed moderate growth-inhibiting activity against *B. subtilis* and *E. coli* but no anti-invasive activity, at least at non-cytotoxic concentrations.

**Scheme 33:**
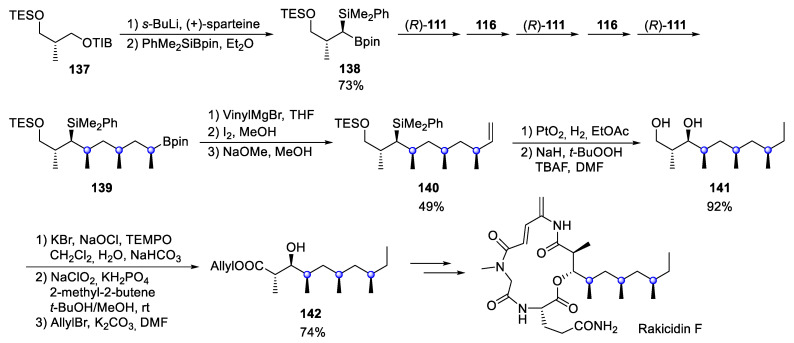
Syntheses of rakicidin F according to Aggarwal et al. [216].

The assembly line strategy was well suited for the construction of the tetramethylated β-hydroxy acid. The benzoate ester **137** required could be produced in three steps from the commercially available (*S*)-Roche ester [216]. Deprotonation in the presence of (+)-sparteine and reaction with PhMe_2_SiBpin provided diastereomerically pure boronic ester **138** [166], which was then fed into the cascade of lithiation-borylation reactions to form **139** (Scheme 33). The olefination of **139** to **140** completed the synthesis of the lipophilic side chain, with a yield of 49% over six steps. The subsequent hydrogenation of the double bond initially caused problems, as Pd/C unexpectedly led to the formation of diastereomers, probably due to the migration of the double bond before hydrogenation. However, this problem could be solved by using PtO_2_. The subsequent Tamao–Fleming oxidation under Woerpel conditions [217] with simultaneous TES deprotection yielded diol **141** in 92% over two steps. Oxidation, followed by allylation, resulted in fragment **142**, which was incorporated into the peptide. Final cyclization gave access to rakicidin F.

## 5. Conclusions

The Matteson homologation, a successive extension of chiral boronic esters, is perfectly suited for the synthesis of complex molecular structures containing many stereogenic centers. The “classical version” allows the introduction of different functional groups in 1,2-*anti*-configuration, but is often limited to this relative configuration, whereby the absolute configuration is determined by the appropriate choice of the chiral auxiliary. The great advantage of the “classical version“ results from the multiple use of the chiral auxiliary, since the successive construction of a growing alkyl chain allows the generation of several stereogenic centers controlled by only one auxiliary. In contrast, in Aggarwal’s lithiation-borylation strategy, new chiral auxiliary reagents must be used for each reaction step, which on the other hand allows the individual introduction of arbitrary configurations of stereogenic centers. Each method has its individual advantages and disadvantages. Depending on requirements, both synthesis protocols are ideally suited for the synthesis of marine natural products.

## Data Availability

The original contributions presented in this study are included in the article.
